# Case Report: A Case of Pediatric Catatonia: Role of the Lorazepam Challenge Test

**DOI:** 10.3389/fpsyt.2021.637886

**Published:** 2021-03-24

**Authors:** Laura Ridgeway, Albert Okoye, Ian McClelland, Dirk Dhossche, Deniz Kutay, Mario Loureiro

**Affiliations:** ^1^Department of Child and Adolescent Psychiatry, University Hospital Waterford, Waterford, Ireland; ^2^Department of Liaison Psychiatry, Children's University Hospital, Dublin, Ireland; ^3^Department of Psychiatry and Human Behaviour, University of Mississippi Medical Center, Jackson, MS, United States

**Keywords:** catatonia, paediatric, depression, lorazepam, catatonia behavior, child

## Abstract

A case of a 12-year-old boy who developed catatonia is presented. He had no previous psychiatric history but has a family history of affective disorder. An extensive medical workup was negative. Despite a negative lorazepam challenge test, lorazepam was titrated up to 24 mg/day, with resolution of most catatonic symptoms. The case highlights an important point in the management of catatonia that may be a source of confusion, i.e., a positive lorazepam challenge test corroborates the diagnosis of catatonia; however, a negative lorazepam challenge test does not negate the diagnosis of catatonia, and subsequent focused benzodiazepine treatment may still be effective.

## Introduction

Catatonia is a potentially life-threatening but treatable condition that could be underdiagnosed and undertreated in children and adolescents (1–3). The prevalence of pediatric catatonia in psychiatric clinics varies from 0.6 to 17% (4, 5). The majority of pediatric catatonic cases occur at pubertal ages (6). In contrast to adults, catatonia in children and adolescents is more common in boys than girls with a ratio of 2:1 (7–9).

For all diagnostic categories of catatonia, the Diagnostic and Statistical Manual of Mental Disorders, Fifth Edition (DSM-5), requires the presence of 3 out of 12 symptoms (10). The presenting symptoms of catatonia are thought to be similar across age ranges. Some symptoms that may be significant in younger patients include refusal to eat or drink, social withdrawal, repetitive movements, and regressive symptoms, such as urinary incontinence (4).

Clinical rating scales originally designed for adults with catatonia have been used in children to assist diagnosis and estimate severity, including the Bush–Francis Catatonia Rating Scale and the Modified Rogers Scale. Cohen also developed a modified Pediatric Catatonia Rating Scale from the Bush–Francis Catatonia Rating Scale (4, 11). A lorazepam challenge test can be considered for diagnostic validation of catatonia (5).

## Case Report

A previously healthy 12-year-old boy (Boy A), presented to the pediatric emergency department with a 4-month history of progressive functional decline and social withdrawal, described by the parents as seeming “lost” and “distant.” He had a brief 2-week period of improvement in his symptoms 2 months previously during holidays from school. His symptoms also seemed to worsen during weekdays and improve over the weekends when he reportedly engaged in sports activity. The authors wondered if this was due to worsening anxiety, low self-confidence, and poor concentration and attention in the increasingly cognitively demanding academic setting. His speech had become minimal with regressed behavior over the preceding 2 weeks, and he required assistance with dressing, washing, feeding, and prompting to void urine.

Boy A was pre-morbidly “a very likable young boy” who enjoyed sports with many friends in school and although could be quiet with a “tendency to be anxious,” he had no prior mental health difficulties. He lived with his mother, father, and two sisters. A recent significant stressor for the family was his father's diagnosis and two admissions in the previous 4 months for bipolar affective disorder following a manic episode. At the time of presentation, his father's mental health was stable.

Apart from a slow gait, the pediatric team's physical and neurological examination was within normal limits. He was admitted to the pediatric medical ward on suspicion of an encephalopathy where he had a comprehensive medical workup, including full blood count, liver function tests, C-reactive protein, thyroid function tests, electrolytes, antistreptolysin-O-titer, lactate dehydrogenase, uric acid, CT scan of the brain, and urine toxicology. All studies were unremarkable.

Child and adolescent psychiatry consult was then sought, and on mental state examination, Boy A appeared preoccupied, anxious, and irritable, with fleeting eye contact. His speech was minimal, and he responded to some questions by shrugging his shoulders, but his engagement improved as the interview progressed. He had apparent difficulty and reluctance to move his limbs, with some evidence of psychomotor slowing. While not initially reporting consistent symptoms of depression, his mood had been pervasively low for more than 2 weeks but no evidence of psychotic symptoms. The initial impression was depression and anxiety with some evidence of psychomotor slowing. Boy A was started on fluoxetine 10 mg OD for 1 week, to be increased to 20 mg OD thereafter, and lorazepam 0.5 mg daily was added to relieve his anxiety and irritability. He was discharged home a few days later, given the stability of his mental state and daily phone reviews, and 1-week psychiatry clinic appointment was agreed.

On review in the clinic, his parents reported reduced oral intake to small sips of fluids, salivating, and inability to swallow oral medication. On urgent readmission to the pediatric medical ward, he showed little reactivity to the environment (stupor) and was essentially mute. There was evidence of psychomotor retardation, catalepsy, waxy, flexibility, rigidity, some degree of posturing, and negativism. He also displayed stereotypies, such as twitching and running his hands over things, mannerisms, and grimacing. His Bush–Francis Catatonia Rating Scale severity score was 30, and screening score was 11, but there was no evidence of psychotic symptoms. His systemic vital signs were within normal limits. His diagnosis was conceptualized as retarded catatonia likely secondary to a depressive illness.

A lorazepam challenge test [2 mg intravenously (IV)] showed no evidence of response. However, the decision was made to continue titrating up the dose of lorazepam while closely monitoring response. Electroconvulsive therapy (ECT) was not deemed an option at that stage as accessing ECT for a pediatric patient in the Republic of Ireland carries many difficulties, including cultural barriers, the necessity of High Court approval, the lack of an experienced center in providing ECT to pediatric patients, and, at the time, a referral to a UK center was complicated by the coronavirus (COVID-19) pandemic. Due to continued refusal to eat, drink, or swallow prescribed fluoxetine after several days of intravenous lorazepam 1 mg TDS, he was started on IV fluids to prevent dehydration.

In addition to the investigations performed on his previous admission, venous blood gas, ammonia, lactate, glucose, creatinine kinase, and cerebrospinal fluid for N-methyl-D-aspartate (NMDA) receptor antibodies were all within normal ranges. MRI scan of the brain and electroencephalogram were also unremarkable. The dietitian started nasogastric feeding after 1 week of no oral intake and 3-kg weight loss, despite then being on lorazepam 10 mg daily in four divided doses. Fluoxetine was then given via the nasogastric tube with daily blood electrolyte monitoring due to risk of refeeding syndrome. Clinically, however, his facial muscles appeared more relaxed and slightly emotionally expressive and he seemed to move his head, arms, and neck somewhat more easily. During physiotherapy sessions over the following few days, he could subsequently stand with assistance, move his arms, and raise both legs at his parent's request. Waxy flexibility became gradually less evident, and he displayed non-verbal interaction with his parents, smiling hesitantly, and engaging in eye contact. Lorazepam had gradually been titrated up to 16 mg daily in four divided doses with no sedation or respiratory compromise by the end of the second week, at which point Boy A could stand up and mobilize independently during physiotherapy sessions. His parents also reported him saying a few words “mum,” “dad,” and he appeared able to swallow a few spoons of food, with noticeably reduced salivation. He had gained 1.5 kg, which increased to 2.7 kg by the end of the third week with improved dietary intake, and nasogastric tube feeds then discontinued.

Although there was still some evidence of psychomotor slowing, rigidity was less evident and his activity was more fluid such that he could now throw, kick, and catch a ball with his parents and was smiling and speaking more frequently. He was however indecisive and appeared “stuck” and required prompting when activity was interrupted. Persistence of these observations informed further increase in the dose of lorazepam to 18 mg by the fourth week of admission, which led to further improvement in psychomotor functioning and physical activity, with still no evidence of sedation. With improvement in psychomotor functioning, however, Boy A became increasingly more tearful and intensely emotional, expressing guilt for relatively minor misdemeanors and repeatedly apologizing to the point of perseveration. He nevertheless was discharged home at the end of the fourth week on lorazepam 18 mg daily and fluoxetine 20 mg daily with a Bush–Francis Catatonia Rating Scale severity score of 3 and screening score of 0. The follow-up plan was for regular phone consultations, weekly psychiatric clinic reviews, and psychology and occupational therapy input.

His emotionality persisted with incessant unprovoked tearfulness and weeping and “a great fear of everything,” including worries that bad things would happen. He was clingy and remained apologetic, repeatedly thanking parents, “thank you so much, you are best mother/father in the world.” He was indecisive and frequently seeking reassurance and permission from parents to engage in even mundane actions such as picking up a ball off the ground while playing. His mother reported some “obsessive–compulsive behavior,” with Boy A insisting that she repeat certain actions and he still showed some motor perseveration, such as, sitting slouched with wrists bent but able to readjust his position when prompted. He, however, no longer displayed purposeless movements.

Lorazepam was consequently increased over a number of weeks to a daily total of 22 mg in four divided doses, and fluoxetine increased to 30 mg OD but without any noticeable change in his depressive symptoms. Our liaison psychiatry colleagues advised a trial of higher doses based on their clinical experience of positive response in a subgroup of adolescents. The decision was made to increase lorazepam to 24 mg daily and fluoxetine 40 mg daily, then 50 mg after 1 week, at which there was a remarkable reduction in emotionality and subsequent complete cessation of tearfulness. Boy A appeared more confident, spontaneous in communication and interaction, and more aware and “awake to his environment.”

He equally, remarkably, actively engaged in outdoor activities, including playing table tennis, soccer, and hurling to high standards with his parents reporting that he was “like a different person on the pitch, much more confident and fast.” There was no evidence of sedation or other side effects from his medication. Boy A maintained his recovery on these doses and was able to return to school 2 months after discharge from the hospital, with lorazepam dose gradually reduced at the rate of 4 mg every 2 weeks but maintained on fluoxetine 60 mg daily.

## Discussion

Schizophrenic disorders are the most common psychiatric disorders associated with catatonia in children and adolescents (7, 8). Mood disorders are the second most common associated psychiatric disorder (4). As somewhat evident in this case, identifiable affective symptoms may only start to emerge after the improvement of catatonic symptoms in some cases (4). Considering Boy A's family history of bipolar affective disorder, this differential cannot be entirely ruled out, and possibly such a diagnosis may become apparent in the future. Traumatic events are also important risk factors for the onset of catatonia in children and adolescents (12). This coincides with the theory that catatonia may represent a primitive evolutionary-based freezing response to a perceived threat (13). In this case, Boy A's father's recent hospitalization and psychiatric diagnosis may represent a traumatic event for Boy A.

Pediatric catatonia is also associated with neurodevelopmental disorders. In a review of six studies, an incidence rate of 4–17% of catatonia was found in adolescents and adults with autistic spectrum disorder (14). Childhood disintegrative disorders, Tourette's syndrome, Down's syndrome, and Prader–Willi syndrome are also associated with higher rates of pediatric catatonia (4, 5). Often, in cases with developmental disorders, the diagnosis can be more difficult due to the overlap of symptoms.

As shown in this case, there is not always a clear identifiable cause initially, and often it is necessary to rule out possible organic causes of pediatric catatonia. An underlying organic condition could be identified in ~20% of pediatric catatonia cases (6, 15). It is important to investigate for these possible contributing conditions, as there are specific treatments for some of them that can improve catatonic symptoms (6, 15). The following medical differential diagnoses are important to consider: epileptic encephalopathy, infections (e.g., viral encephalitis, typhoid fever, toxoplasmosis), autoimmune diseases (e.g., neuropsychiatric systemic lupus erythematosus and anti-NMDA receptor encephalitis, pediatric autoimmune neuropsychiatric disorders associated with streptococcal infections (PANDAS), encephalopathy associated with autoimmune thyroid disease), toxic-induced states (e.g., lithium, ecstasy), and many metabolic and genetic disorders (4, 5, 8, 15).

Pediatric catatonia usually has an acute onset, but its onset can also be gradual. Duration can be transient or chronic for weeks or months (4). Children with catatonia are at risk of complications secondary to akinesia, such as pneumonia, malnutrition, dehydration, contractures, decubitus ulcers, or thrombosis (4). Catatonia has the potential to progress to malignant catatonia if left untreated. The use of antipsychotic medication can increase the risk of progression to malignant catatonia (2, 8). Malignant catatonia is a potentially lethal condition. In this severe form, an exacerbation of motor and non-motor catatonic symptoms is accompanied by systemic symptoms, such as fever, autonomic instability, and delirium (2, 4, 8).

As in adults, the first-line treatment in pediatric catatonia should be benzodiazepines (lorazepam), given IV or intramuscularly (1, 4, 5, 16). In most cases, symptoms improve considerably after lorazepam challenge with 1–2 mg (4). If a positive response is observed, it is then assumed to be catatonia and the dose titrated further for optimal response and maintenance of improvement, often to between 10 and 20 mg per day (4, 5). It is worth noting that the authors made a focused effort to continue with lorazepam despite a negative challenge test for reasons aforementioned and evidence for its effectiveness in the age group. In a prospective study of children and adolescents, benzodiazepines were effective in 65% of 66 patients (16). The most common side effect observed was excessive sedation (16). It is also imperative to observe closely for signs of respiratory depression.

Although not required in this case, ECT would be the second-line treatment if benzodiazepines were not effective or in severe cases such as malignant catatonia (17). The response rate for ECT in catatonia in youths is 76–92% (18, 19). However, there can be logistic and cultural barriers to using ECT in pediatric patients.

Importantly, associated psychiatric or medical conditions may also require separate treatment in addition to the treatment of catatonic symptoms outlined. The authors in this case observed a temporal relationship between administration of high-dose fluoxetine and dramatic improvement in depressive symptoms. It is however not completely clear if this effect was synergistic with lorazepam, as existing literature appears not to support a role for fluoxetine in the treatment of catatonia. Further case reports might be helpful in this regard.

## Data Availability Statement

The original contributions presented in the study are included in the article/Supplementary Material, further inquiries can be directed to the corresponding author/s.

## Ethics Statement

Written informed consent was obtained from the minor(s)' legal guardian/next of kin for the publication of any potentially identifiable data included in this article.

## Author Contributions

LR wrote case report and article. AO was the lead treating clinician for this case and reviewed the case report and article as senior author. IM, DD, DK, and ML provided clinical advice to AO on the management of the case. All authors contributed to the article and approved the submitted version.

## Conflict of Interest

The authors declare that the research was conducted in the absence of any commercial or financial relationships that could be construed as a potential conflict of interest.

## References

[1] Sorg EM Chaney-Catchpole M Hanzen EP. Pediatric catatonia:a case series-based review of presentation, evaluation, and management. Psychosomatics. (2018) 59:531–8. 10.1016/j.psym.2018.05.012 30104020

[2] Cornic F Consoli A Tanguy ML Bonnot O Perisse D Tordjman S. Association of adolescent catatonia with increased mortality and morbidity: evidence from a prospective follow-up study. Schizophr Res. (2009) 113:233–40. 10.1016/j.schres.2009.04.021 19443182

[3] Ghaziuddin N Dhossche D Marcotte K. Retrospective chart review of catatonia in child and adolescent psychiatric patients. Acta Psychiatr Scand. (2012) 125:33–8. 10.1111/j.1600-0447.2011.01778.x 22040029

[4] Benarous X Raffin M Ferrafiat V Consoli A Cohen D. Catatonia in children and adolescents: new perspectives. Schizophr Res. (2018) 200:56–67. 10.1016/j.schres.2017.07.028 28754582

[5] Dhossche DM Goetz M Gazdag G Sienaert P. New DSM-5 category 'unspecified catatonia' is a boost for pediatric catatonia: review and case reports. Neuropsychiatry. (2013) 3:401–10. 10.2217/npy.13.42

[6] Consoli A Raffin M Laurent C Bodeau N Campion D Amoura Z. Medical and developmental risk factors of catatonia in children and adolescents: a prospective case-control study. Schizophr Res. (2012) 137:151–8. 10.1016/j.schres.2012.02.012 22401837

[7] Cohen D Nicolas JD Flament MF Perisse D Dubos PF Bonnot O. Clinical relevance of chronic catatonic schizophrenia in children and adolescents: evidence from a prospective naturalistic study. Schizophr Res. (2005) 76:301–8. 10.1016/j.schres.2005.01.014 15949662

[8] Takoaka K Takata T. Catatonia in childhood and adolescence. Psychiatr Clin Neurosci. (2003) 57:129–37. 10.1046/j.1440-1819.2003.01092.x 12667158

[9] Cohen D Flament M Dubos PF Basquin M. Case series: catatonic syndrome in young people. J Am Acad Child Adolesc Psychiatry. (1999) 38:1040–6. 10.1097/00004583-199908000-00021 10434497

[10] American Psychiatric Association. Diagnostic and Statistical Manual of Mental Disorders: DSM-5. 5th ed. Washington, DC: American Psychiatric Publishing (2013). 10.1176/appi.books.9780890425596

[11] Benarous X Consoli A Raffin M Bodeau N Giannitelli M Cohen D. Validation of the Pediatric Catatonia Rating Scale (PCRS). Schizophr Res. (2016) 176:378–86. 10.1016/j.schres.2016.06.020 27377978

[12] Dhossche DM Ross CA Stoppelbein L. The role of deprivation, abuse, and trauma in pediatric catatonia, without a clear medical cause. Acta Psychiatr Scand. (2012) 125:25–32. 10.1111/j.1600-0447.2011.01779.x 22017207

[13] Moskowitz AK. “Scared stiff”: catatonia as an evolutionary-based fear response. Psychol Rev. (2004). 111:984–1002. 10.1037/0033-295X.111.4.984 15482070

[14] Dhossche DM Shah A Wing L. Blueprints for the assessment, treatment and future study of catatonia in autism spectrum disorders. Int Rev Neurobiol. (2006) 72:267–84. 10.1016/S0074-7742(05)72016-X 16697303

[15] Lahutte B Cornic F Bonnot O Consoli A An-Gourfinkel I Sedel F. Multidisciplinary approach of organic catatonia in children and adolescents may improve treatment decision making. Prog Neuro-Psychopharmacol Biol Psychiatr. (2008) 32:1393–8. 10.1016/j.pnpbp.2008.02.015 18417262

[16] Raffin M Zugaj-Bensaou L Bodeau N Milhiet V Laurent C Cohen D. Treatment use in a prospective naturalistic cohort, of children and adolescents with catatonia. Eur Child Adolesc Psychiatr. (2015) 24:441–9. 10.1007/s00787-014-0595-y 25159089

[17] Cohen D Paillere-Martinot ML Basquin M. Use of electroconvulsive therapy in adolescents. Convulsive Therapy. (1997) 13:25–31. 9152585

[18] Consoli A Benmiloud M Wachtel L Dhossche D Cohen D Bonnot O. Electroconvulsive therapy in adolescents with the catatonia syndrome: efficacy and ethics. J ECT. (2010) 26:259–65. 10.1097/YCT.0b013e3181fb3924 21099377

[19] Grover S Malhotra S Varma S Chakrabarti S Avasthi A Mattoo SK. Electroconvulsive therapy in adolescents: a retrospective study from north India. J ECT. (2013) 29:122–6. 10.1097/YCT.0b013e31827e0d22 23296394

